# The Effects of an Ecological Diversifying Experience on Creativity: An Experimental Study

**DOI:** 10.3389/fpsyg.2020.01396

**Published:** 2020-07-14

**Authors:** Alice Chirico, Sofia Carrara, Sofia Bastoni, Elena Gianotti, Andrea Gaggioli

**Affiliations:** ^1^Università Cattolica del Sacro Cuore di Milano, Milan, Italy; ^2^Department of Psychology, Università Cattolica del Sacro Cuore di Milano, Milan, Italy; ^3^Applied Technology for Neuro-Psychology Lab, IRCCS Istituto Auxologico Italiano, Milan, Italy

**Keywords:** creativity, diversifying experience, cognitive flexibility, divergent thinking, Torrance test, dialogue in the dark, schema violation

## Abstract

Sometimes, life houses rare and unexpected events, such as moving abroad or meeting a special person unexpectedly. Recently, these situations have been indicated as “diversifying experiences” (DEs), defined as unusual and unexpected events that drag people outside their daily routine and accustomed schemas. The core mechanism of DEs would entail the disruption of our mental schema, which can facilitate unexpected connections among even distant ideas, thus enhancing people’s cognitive flexibility, that is, a key component of creative thinking. Despite both qualitative and lab-based studies have investigated the features of these experiences, an ecological assessment of their properties also in relation with creativity is still an open issue. The aim of this research is to study the DE–creativity link in a more ecological way, on the basis of a real-life disruptive experience of light deprivation. Specifically, we compared an ecological DE artistic established entertainment format (i.e., “dialogue in the dark,” which is seeing people perform several daily life activities but in the absence of light) with an equivalent experience in which the same activities were acted in the sunlight. The absence of light played the role of violating mechanism, framed within the ecological experiential format of the “dialogue in the dark.” We compared visitors’ emotional profile [Positive and Negative Affect Schedule (PANAS), *ad hoc* Adjective Checklist], perceived impact of the experience [Centrality of Event Scale (CES)], and creative performance [Torrance Tests of Creative Thinking (TTCT)] in both groups of sighted people (in absence of light vs. in presence of light); and we also controlled for people’s openness to experience and need for cognitive closure, as dispositions. Results showed that (vs. control group) “dialogue in the dark” (i) led to worse creative performances, (ii) produced more intense positive affect, and (iii) resulted as a more impacting experience. Intense short-term impact of DE could have been detrimental for participants’ creativity. People may need more time to elaborate the DE and accommodate existing schema to generate more creative ideas. This is the first study proposing and succeeding in demonstrating the feasibility to investigate even real complex DEs in a controlled way, thus outlining how their link with creativity can take place in real life.

## Introduction

As defined by Ritter et al. (2012) and Damian and Simonton (2014), diversifying experiences (DEs) involve unusual, unexpected and disruptive events, different from our daily routine; thus, they are able to drag people outside their “realm of normality” (Damian and Simonton, 2015). With this regard, all DEs would share a common underlying mechanism entailing a violation of our accustomed mental structures, which people use to process the complexity of the environment and to generate predictions concerning the outside world (Gocłowska et al., 2014). Mental schema are abstract generalizations of information at the base of people’s expectancies toward the world, themselves, and the others, which are crucial to orient our behavior in the world (Roese and Sherman, 2007). Phylogenetically, approaching and exploring unpredictability by overcoming accustomed mental schema have been a highly desirable functional ability (Gocłowska et al., 2017). For example, our ancestors’ preference for novelty and surprise encouraged them to explore the world and to discover new habitats and foods. Indeed, the pursuit and acceptance of unusual ideas have led to the development of pioneering innovations that required innovators to go against their knowledge and violate their assumptions about the world (Gocłowska et al., 2017).

With this regard, the main potential of schema violations as a core component of DEs consists in creating a bridge between these unusual experiences and creativity (Damian and Simonton, 2014) or, at least, between DEs and a specific component of creative thinking (Ritter et al., 2012), which is cognitive flexibility (Oztop, 2017). Cognitive flexibility can be defined as the ability of our cognitive system to adapt to changes by shifting the focus of attention, formulating new action plans and new states of activation, and modifying internal cognitive processes (Deak, 2003). Damian and Simonton (2014) and more recently, Gocłowska et al. (2018) compared DE with stressful events, and they elaborated that DE–creativity link should be depicted as an inverted U shape, where moderate levels of novelty and unconventionality stemming from a DE could result into increased creative thinking. In other terms, to maximize the potential of DEs for enhancing creative thinking, the perceived intensity of DE should be moderate, stimulating people’s resources instead of just exploiting them. Therefore, not all DEs can be conductive to creativity.

With this regard, empirical evidences, albeit heterogenous, have investigated specific conditions leading to a link between DEs and creativity (for a review, see Damian and Simonton, 2014) or more specifically, between schema violations and cognitive flexibility (e.g., Ritter et al., 2012) as a core component of creativity thinking process (Torrance, 1969). Particularly, it would be possible to identify specific approaches to the study of DEs and creativity. Each of them either scaled up DE by conceiving it as complex experience (*macrolevel of analysis*) with its subcomponents or scaled down DE by manipulating specific schema violation (*microlevel of analysis*).

### A Macrolevel of Analysis: Diversifying Experiences as Complex Experiences

Damian and Simonton (2014) assumed that the link between a complex DE and creativity can be nurtured by seeing things in an unconventional way, thus leading people to imagine the impossible. They suggested that this process would lead to new cognitive paths, as a source of mental flexibility (Simonton et al., 1999). DEs can include, but are not limited to, biculturalism, multilingualism or bilingualism, psychopathology, and familiar unpredictability (Damian and Simonton, 2014). Correlational, historiometric, and psychometric studies evidenced a link between these special types of DEs and with both exceptional (Big-C) and everyday creativity (little-c) (Damian and Simonton, 2014). For instance, multiculturalism was beneficial for little-c creativity only if people were high in openness to experience (Leung and Chiu, 2008). It maybe that individuals high in this trait would have also a higher predisposition to deal with different ideas or perspectives at the same time, thus overcoming initial strain related to managing diversity. Actually, diversity acts as a facilitating factor for cognitive flexibility and creativity at general, not only at the individual level but also at the group level (Cox and Blake, 1991; McLeod et al., 1996). However, group members should be willing to adopt other members’ diverse perspective and should be guided by beliefs in favor of diversity instead of supporting a cognitive attitude toward similarities (Hoever et al., 2012). In other terms, a certain amount of openness toward diversity was needed also at the group level to lead teams toward better creative performances and a more flexible style of thinking.

### A Microlevel of Analysis: The Role of Schema Violation

If is largely accepted that that schema violation acts as a central factor in DE–creativity link, it is crucial to outline empirically how they can modulate this relationship. With this regard, DEs have been scaled down at the level of specific schema violations reproduced in the lab. Laboratory studies provide conflicting results showing that cognitive violation not always leads to a better creative performance. For instance, Ritter et al. (2012) used simulations of situations that violated the logic of physics (e.g., a fallen glass lifting instead of shattering into thousand pieces) involving participants actively [through a virtual reality (VR) simulation] or passively (through a movie). Results revealed that when individuals were actively involved into the violation, their cognitive flexibility increased significantly than when they watched only the movie. This can suggest that even small-scale violations could “open people’s mind,” connecting ideas that were previously far apart. This happened also when individuals were not directly engaged into specific paradoxical action plans but when they identified with other individuals realizing paradoxical actions (Ritter et al., 2014).

However, when individuals actively interacted with another person in the lab, who violated their stereotype-related schema, and their psychophysiological reaction was primarily of threat, subsequent affect associated with the human actor was more negative and creative performance worsened (Mendes et al., 2007). Schema violation and creativity link did not hold under all circumstances, especially if there was an involvement with a real person and not just a simulation of it (e.g., VR) and when specific schema was violated.

With this regard, according to the most recent model on DEs (Gocłowska et al., 2018), appraisals activated by individuals to process the schema violation stemming from a DE should play a central role in facilitating (or not) creativity. Indeed, depending on the evaluation of schema violation, individuals’ general attitude toward a DE would change significantly, either as a threat or as a challenge. Only the latter positive attitude (i.e., challenge) should be related to higher creative flexibility. With this regard, two key appraisals should be at the base of a positive evaluation of a violation, that is, surprise and interest (Gocłowska et al., 2017). Both are epistemological emotions (Silvia, 2010), but surprise would act as a potential trigger of interest, which would be directly responsible for a positive evaluation of a given violation. Therefore, being surprised in front of something able to challenge our expectancies should be not enough to evaluate the source as positive. Being motivated to look for more information, connecting even distant ideas and process more data should be the key. Whereas being high in openness to experience personality trait should facilitate an appraisal of interest, being high in the need for structure, cognitive closure, and fixed rules would be detrimental (Gocłowska et al., 2017). Crucially, people with a higher need for fixed structure, hardly benefit from schema violations and showed worse creativity self-efficacy and creative thinking abilities (Gocłowska et al., 2017). Finally, besides the mere cognitive aspects, also the role of the body emerged as a key variable determining whether a violation would be either conductive to cognitive flexibility or not (Huang and Galinsky, 2011). This may suggest that even realistic VR simulation can lack of important body-related components that should be considered in the equation of DE–creativity link.

#### This Study: The Dialogue in the Dark Experience

If the existence of a link between DEs and creativity both at a macrolevel and a microlevel of analysis has been accepted, evidence still show inconsistencies due to the type of DEs and violations considered, the low control of correlational and psychometric research, and the high control but low degree of ecological validity of current experimental manipulations of DEs.

VR simulations provided by Ritter et al. (2012, 2014) provided crucial initial evidence on the role of realistic basic schema violations without social interactions – disembodied experiences – on creative thinking but are still far from the real occurrence of DE in reality. Both scaling up DEs as complex experiences and scaling down them as schema inconsistencies reproduced in the lab could hinder a full comprehension of this phenomenon in real life (Damian and Simonton, 2014).

To address this issue, we built upon existing theoretical and experimental evidence on DE to advance the knowledge on how real DEs would impact individuals’ creativity. This allowed for enhancing ecological validity while maintaining a controlled setting. We identified an artistic established format composed by a set of real-life group activities to be performed in the absence of light, which is called “dialogue in the dark,” as a potentially diversifying and ecological experience.

“Dialogue in the dark” violates people’s most basic expectancies regarding light, which is a primary source of information in our daily life. Sighted people heavily rely on vision to represent their peri-personal space. On the contrary, blind people use the haptic system (Postma et al., 2007). What would it be like to live as a blind person for some time? This artistic format answers this question. “Dialogue in the dark” violates sighted people’s typical ways of representing space, forcing them to activate an alternative system (haptic system instead of vision) (Postma et al., 2007).

This study can advance current paradigms in the study of DEs at two levels. First, it can provide an ecological setting resembling real-life DE. Then, it can allow observing the effect of this experience of people’s creativity immediately after its occurrence in a controlled setting.

## Materials and Methods

### Research Design, Measures, and Instruments

This research is composed of two studies. In both studies, before signing the informed consent, participants were fully briefed about the research purpose and were informed that they could take part on a research conducted by the Università Cattolica del Sacro Cuore in collaboration with the Institute of the Blinds. They were also told that they were free to leave the study at any time. The experimental protocol was approved by the Ethical Committee of the Università Cattolica del Sacro Cuore prior to data collection. Each participant provided written informed consent for study participation. The whole procedure was carried out in accordance with the Declaration of Helsinki.

In Study 1, the “diversifying” nature of the “dialogue in the dark” was measured following the guidelines provided by the reference framework on DEs (Ritter et al., 2012; Damian and Simonton, 2014; Gocłowska et al., 2018).

Specifically, “dialogue in the dark” diversifying nature was measured by means of the following:

(i) *Ad hoc* Adjective Checklist. This self-reported instrument was designed *ad hoc* for this study to measure the diversifying potential of the “dialogue in the dark.” The scale was created from the adjectives that Ritter et al. (2012) and Damian and Simonton (2014) attributed to DEs. The adjectives that constitute the scale were unusual, unexpected, engaging, ordinary, and intense. Participants were asked to indicate their degree of agreement with the adjectives listed in 5-point Likert response scale format (1 = not at all; 5 = at all). Because Cronbach alpha was.71, we chose to consider each item separately.
(ii) Centrality of Event Scale (CES). According Gocłowska et al. (2018), DEs require individuals to cope and adapt; thus, they are comparable with stressful events. The long 20-item version of the CES was composed of three dimensions on a 5-point Likert scale from 1 (strongly disagree) to 5 (totally agree), measuring the extent to which the memory of the event becomes (a) a reference point in everyday life, (b) a key component of personal identity, and (c) a turning point in the personal life story (Berntsen and Rubin, 2006; Ionio et al., 2018). To be more conservative, we both aggregated the scores to obtain a global CES score, and we also computed specific analyses for each single item (subdimension) of the scale: (i) “I feel that this event has become part of my identity” (label: “Part of identity”); (ii) “This event has become a reference point for the way I understand myself and the world” (label “Comprehension”); (iii) “I feel that this event has become a central part of my life story” (label: “Central in life”); (iv) “This event has colored the way I think and feel about other experiences” (label: “Other experiences”); (v) “This event permanently changed my life” (label: “Life change”); (vi) “I often think about the effects this event will have on my future”; and (vii) “This event was a turning point in my life” (label: “Turning point”). The CES (α coefficient = 0.88) was selected to assess the personal impact of DE as a potentially stressful event (Folkman and Lazarus, 1984). In Study 1, this scale was assessed after 2 weeks of the “dialogue in the dark” experience.

In Study 2, a comparison between the “dialogue in the dark” and an equivalent experience that consists of the same set of activities preformed in the presence of light was carried out (control condition). This control experience was as much similar as possible – for content and duration – to the experimental one. “Dialogue in the dark” and control experience differed regarding the presence (vs. absence) of light. In the “dialogue in the dark,” participants performed a set of daily activities in the absence of light. Conversely, the control condition consists of the same set of activities performed in a park in the presence of light.

Specifically, dialogue in the dark is an experience in the dark, where blind guides lead visitors in small groups (eight people with normal or corrected sight persons, in this study) through different settings, that is, playing with a ball together, walking on the grass, touching the water of a little lake, tasting some spices, and so forth, in the absence of light.

The control group, instead, lived an equivalent experience in Parco Sempione (a renowned park in Milan), which was equivalent in duration and content to the “dialogue in the dark” but more “ordinary” because the same daily activities were done in the presence of light. At the end of both the experiences, a debriefing procedure was carried out in a pub, so that participants could talk about the experience they just lived.

Study 2 followed a *between-subjects* design, where participants were assigned only to the “dialogue in the dark” (experimental) or to the control condition.

Immediately after both experiences, participants were required to complete the *ad hoc* Adjective Checklist and the CES, again, as well as the following self-reported instruments:

(i) Creative thinking. Participants completed the Italian version of subtest 5 (i.e., unusual uses of a box) of the Torrance Tests of Creative Thinking (TTCT) (Torrance, 1974; Torrance et al., 1989). This subtest was scored according to Guilford’s divergent thinking factors: fluency (i.e., number of relevant responses), elaboration (i.e., the number of details in the answers), originality (i.e., statistically infrequent but relevant answers), and flexibility (i.e., the number of different categories within relevant responses) (Guilford, 1950, 1959, 1967). The “Unusual Uses” task lasted 10 min, and participants were tasked to generate as much solutions as possible concerning interesting and unusual ways of using a cardboard box.

Instructions are reported as follows:

“Almost everyone is used to throw away used cardboard boxes, yet there are thousands interesting and unusual ways of using them. In the lines of the current page and the following one, please, make the longest list of interesting and unusual ways of using the cardboard boxes that you can imagine. Do not think only about boxes of particular size. You can use all kinds of boxes you want. Do not limit yourself to the uses you have seen or heard before. Try to imagine as many new uses as possible.” With every measure, “the instructions are designed to motivate respondents to give unusual, detailed responses” (Cramond et al., 2005; p. 284). Torrance based TTCT’s scoring on Guilford’s divergent thinking factors: fluency, which refers to the number of relevant responses; flexibility, which is the number of different categories within relevant responses; originality, which refers to statistically infrequent but relevant answers; and elaboration, which refers to the number of details in the answers (Cramond et al., 2005). Here, we involved two independent raters to score participants’ performances at subtest 5 for fluency, flexibility, originality, and elaboration, achieving good levels of reliability (Rater 1 and Rater 2: Cronbach α = 0.80).

(ii) The Positive and Negative Affect Schedule (PANAS). The Italian version of the PANAS (Terraciano et al., 2003) [α coefficients: 0.86–0.90 for positive affect (PA) and 0.84–0.87 for negative affect (NA)] was used. The scale measures PA and NA states at a certain time. It is composed of 10 adjectives indicating PAs and the other 10 referring to NAs. Participants had to indicate how much the adjectives described how they felt in that moment – after the “dialogue in the dark”/control experience – by using a 5-point Likert scale. This scale combined with the *ad hoc* Adjective Scale allowed assessing participants’ emotional experience. Gocłowska et al. (2018) suggested that only when a DE avoids a negative appraisal of threat could better creative abilities be achieved.

Finally, following up main findings on schema-violation effectiveness for enhancing creative thinking (e.g., Huang and Galinsky, 2011; Gocłowska et al., 2014, 2017), also participant’s openness to experience and need for closure were measured and included as covariates. Specifically, participants completed the following:

(i) The Openness to Experience Scale. The *Openness to Experience Scale* (α coefficient = 0.80) was used to measure participants’ mental openness. This is a subscale of the Italian version of the Big Five Inventory (Ubbiali et al., 2013), selecting only those items tapping into the construct of Openness to Experience (10 items). Example items are “I am original, come up with new ideas.” Participants were asked to complete the scale by using a 5-point Likert response scale.
(ii) The Need for Cognitive Closure Scale. The *Need for Cognitive Closure Scale* (Pierro et al., 1995) (α coefficient = 0.84) was used to detect the degree of personal need for structure of participants. The scale consists of 42 items relating to five main dimensions of the need for cognitive closure, namely, (a) need for order, that is, the need for structuring in one’s environment; (b) intolerance for ambiguity, which refers to the emotional discomfort produced by living ambiguous situations; (c) decision making, which concerns the need to quickly get to a conclusion; (d) mental closure, as the tendency to prevent one’s knowledge from being questioned by different or conflicting opinions; and (e) need for predictability, that is, the desire to have safe and generalizable knowledge, ensuring a sure predictability of the contexts in which one will be operating (Pierro et al., 1995). Participants were asked to express their agreement with all statements on a 7-point Likert scale.

### Data Analysis

Two normality tests (i.e., Kolmogorov–Smirnov and Shapiro–Wilk) were carried out to determine if variables were normally distributed.

For Study 1, this did not have implications, because we relied only on descriptive statistics of affect (PANAS) and personal impact of the experience (CES).

In Study 2, because almost all variables were not normally distributed, first, intercorrelations among raters’ creativity-dimensions scores were carried out by computing Spearman’s rho coefficients. Then, a Mann–Whitney test for all variables was carried out, in order to test the effect of the “dialogue in the dark” (vs. control group) on creative thinking dimensions, affect, and personal impact of the experience.

Finally, a series of analysis of covariance (ANCOVA) with “group” (“dialogue in the dark” vs. control) as independent variable, Global CES score as measure, and all dimensions of the Openness to Experience and the Need for Cognitive Closure Scale as covariates were carried out.

All statistical analyses were performed using IBM SPSS Statistics software (Version 21, release 21.0.0.0 64 bit edition).

### Study 1

#### Aim

The aim of this preliminary study was to measure the affective profile of the “dialogue in the dark” immediately at the end of the experience and its personal impact after 2 weeks.

#### Participants and Procedure

We conducted Study 1 with a separate group of volunteers, in order to test the extent to which the “dialogue in the dark” could be considered as a DE. Participants in Study 1 (*N* = 30; 19 females) were all sighted adults with mean age = 30.23 (*SD* = 10.08). They were recruited locally (i.e., at the Institute of the Blinds of Milan) among people willing to try the “dialogue in the dark” experience.

The procedure is composed of three steps: (i) participants were briefed on the aim of the study and were asked for their informed consent. (ii) Participants took part of the “dialogue in the dark” experience; participants answered the *ad hoc* Adjectives Scale immediately after the “dialogue in the dark” experience. (iii) Participants filled out the CES short Italian version (Ionio et al., 2018) – sent to them through email – 2 weeks after the experience, in order to measure the long-term impact of the “dialogue in the dark.”

### Results

Descriptive statistics showed that “dialogue in the dark” was a highly unusual, unexpected, involving, and intense but less ordinary experience [Unusual (mean = 4.1; *SD* = 1.21); Unexpected (mean = 3.57; *SD* = 1.16); Involving (mean = 4.93; *SD* = 0.25); Ordinary (mean = 1.33; *SD* = 1.06); and Intense (mean = 4.87; *SD* = 0.35), within a range from 1 to 5].

Personal impact of the experience after 2 weeks resulted as moderate: Part of identity (mean = 3.17; *SD* = 0.98); Comprehension (mean = 3.39; *SD* = 1.08); Central in life (mean = 2.56; *SD* = 1.12); Other Experiences (mean = 3.13; *SD* = 1.01); Life change (mean = 2.91; 1.04); Effects on future (mean = 2.7; *SD* = 1.02); and Turning point (mean = 2.53; *SD* = 1.04).

### Study 2

#### Aim

The aim of this experimental study was to test whether an ecological DE based on clear violation of light deprivation, as the “dialogue in the dark,” which follows a codified format, could significantly enhance participants’ creative thinking abilities and lead to a higher PA and higher personal impact as compared with an equivalent control experience.

#### Participants and Procedure

Participants in this study (*N* = 133) were all sighted adult volunteers: 71 (31 males) with mean age 32.5 years (*SD* = 11.4) were in the experimental group, whereas 62 (30 males) with mean age 30.9 (*SD* = 11.6) were in the control one. There was no significant difference in the openness to experience disposition between the experimental group (mean = 3.26; *SD* = 0.475) and the control one (mean = 3.31; *SD* = 0.484). Moreover, participants of both groups did not significantly differ regarding all subdimensions of the Need for Closure scale: (i) *Decision Making*, experimental group (mean = 28; *SD* = 5.06) versus control group (mean = 27.6; *SD* = 4.47); (ii) *Need for Order*, experimental group (mean = 38.6; *SD* = 7.47) versus control group (mean = 38.8; *SD* = 7.51); (iii) *Need for Predictability*, experimental (mean = 27.9; *SD* = 4.34) versus control (mean = 27.5; *SD* = 4.19); (iv) *Intolerance for Ambiguity*, experimental (mean = 32.4; *SD* = 6.79) versus control (mean = 34.2; *SD* = 8.37); and (v) *Mental Closure*, experimental (mean = 30.3; *SD* = 4.33) versus control (mean = 30.1; *SD* = 4.86). Participants in the control group were recruited through social media, and participants in the experimental group were recruited where the experience of the “dialogue in the dark” took place (i.e., Institute of the Blinds of Milan).

After participants in both the control and experimental groups read and signed the informed consent, they were asked to complete the Openness to Experience Scale and the Need for Cognitive Closure Scale. Then, the experimental group lived the “dialogue in the dark,” whereas participants in the control group were recruited at the place in which this control experience was realized (in a park called Parco Sempione). Both experiences lasted around 1 h 15 min.

At the end of both experiences, all participants were asked to perform the same verbal form of the Torrance Tests “unusual uses for a cardboard box” (Torrance, 1966) immediately after the experience. After that, participants completed the Adjective Scale created *ad hoc* for validating the “dialogue in the dark” as a DE and the CES short Italian version (Ionio et al., 2018) again, and the PANAS (Terraciano et al., 2003), measuring affect.

### Results

#### Creative Thinking

Spearman’s correlations between rater 1 and rater 2’ fluidity, flexibility, originality, elaboration scores, were computed to check for raters’ level of agreement. Since their agreement was high, we proceeded by aggregating the scores of Rater 1 and Rater 2 for each of the creative thinking dimensions (i.e., fluidity, flexibility, originality and elaboration) (see Table 1). A Mann–Whitney test was used comparing each group (“dialogue in the dark” vs. control) regarding fluidity, flexibility, originality, and elaboration dimensions. Results showed a significant effect of the group on the scores of all the dimensions. Comparing experimental and group’s means, we found out that the experimental group performed significantly worse than the control group in all the four dimensions: fluidity, flexibility, originality, and elaboration (see Table 2).

**TABLE 1:** Spearman’s correlations between rater 1 and rater 2 scores.

Rater 2	Rater 1			

Creativity dimensions	Fluidity	Flexibility	Originality	Elaboration
Fluidity	0.886**	0.847**	0.822**	0.183**
Flexibility	0.845**	0.870**	0.760**	0.172**
Originality	0.850**	0.800**	0.828**	0.077**
Elaboration	0.185**	0.158**	0.171**	0.591**

* = *p* < 0.05; ** = *p* < 0.001. *In bold are all correlation coefficients on the same dimension of creative thinking.*

**TABLE 2:** Mann–Whitney test with group as independent variable; and fluidity, originality, and elaboration as dependent variables.

Creativity dimension	Experimental		Control		Mann–Whitney	
			
	Mean	Mdn	Mean	Mdn	Sign.	*U*
Flexibility	8.29	8	10.22	10	**0.006**	2,795.5
Fluidity	12.29	10.5	18.45	16.5	**<0.001**	3,088.5
Originality	10.96	10	16.07	14.75	**0.001**	2,956
Elaboration	1.81	1	3.77	2.5	**0.001**	2,886

*In bold are all significant values.*

#### Diversifying Nature of “Dialogue in the Dark” Versus Control Condition

Scores at the *ad hoc* Adjective Scale showed that the experimental group evaluated the “dialogue in the dark” experience as significantly more unusual, unexpected, involving, and intense than did the control group (Table 3).

**TABLE 3:** Mann–Whitney test and group statistics with group as independent variable and Adjective Scale items as dependent variables.

Adjectives	Experimental		Control		Mann–Whitney	
			
	Mean	Mdn	Mean	Mdn	Sign.	*U*
Unusual	4.42	5	3.98	4	0.009	1,409
Unexpected	3.94	4	3.42	4	0.012	1,411.5
Involving	4.84	5	4.32	4	0.000	1,156
Ordinary	1.31	1	1.88	2	< 0.001	2,530
Intense	4.67	5	3.73	4	< 0.001	754.5

*Maximum score was 5.*

#### Personal Impact of “Dialogue in the Dark” Versus Control Condition

Personal impact of “dialogue in the dark” versus control condition resulted as significantly higher at the level of single items of CES but not for the global score (see Table 4).

**TABLE 4:** Mann–Whitney test and group statistics with group as independent variable and Centrality of Event Scale’s items as dependent variables.

Personal impact	Experimental		Control		Mann–Whitney	
			
	Mean	Mdn	Mean	Mdn	Sign.	*U*
Part of identity	4.38	4.5	3.56	3	**0.002**	1,280.5
Comprehension	4.56	4	3.93	4	**0.037**	1,483.5
Central in life	3.77	4	2.98	3	**0.003**	1,318.5
Marking event	4.56	5	4.14	4	**0.109**	1,577
Life change	4.56	5	3.22	3	**<0.001**	1,027.5
Effects on future	4.28	4	3.92	4	**0.322**	1,695
Turning point	3.61	4	2.76	3	**0.005**	1,339
Global score of CES	26.1	29.0	24.1	23.0	0.066	1,783

*In bold are all significant values. *Maximum score was 7. (i) “I feel that this event has become part of my identity” (label: “Part of identity”); (ii) “This event has become a reference point for the way I understand myself and the world” (label “Comprehension”); (iii) “I feel that this event has become a central part of my life story” (label: “Central in life”); (iv) “This event has colored the way I think and feel about other experiences” (label: “Other experiences”); (v) “This event permanently changed my life” (label: “Life change”); (vi) “I often think about the effects this event will have on my future” (label: “Effect on Future”); and (vii) “This event was a turning point in my life” (label: “Turning point”).**

#### Affective Profile of “Dialogue in the Dark” and Control Condition

The experimental group experienced significantly more intensive PA than the control group, whereas there were no differences regarding global NA (see Table 5).

**TABLE 5:** Mann–Whitney test and group statistics with group as independent variable and PANAS items as dependent variables.

Items	Experimental		Control		Mann–Whitney	
			
	Mean	Mdn	Mean	Mdn	Sign.	*U*
Attentive	4.29	4	3.88	4	0.006	1,380.5
Enthusiastic	4.29	4	3.76	4	0.001	1,258.5
Concentrate	4.08	4	3.45	3.5	<0.001	1,118
Nervous	1.52	1	1.90	2	0.015	2,253
Jittery	1.56	1	1.82	2	0.047	2,212.5
Excited	3.19	3	2.78	3	0.046	1,485.5
Irritable	1.54	1	1.75	1.5	0.041	2,173
Positive Affect	37.92	38	35.52	36	0.020	1,356

*Maximum score was 5. Only significant results were reported. PANAS, Positive and Negative Affect Schedule.*

#### Potentially Intervenient Factors

We conducted an ANCOVA to control for the potential influence of “openness to experience” and “need for structure” on DE–creativity link. Few significantly results were found.

A one-way ANCOVA was conducted to determine statistically significant differences between the experimental group and the control one on the CES items and CES global score, after controlling for the dispositional variables of Openness to Experience and Need for Cognitive Structure. Results showed that – after controlling the Openness to Experience Scale’s global score – there was a significant effect of groups on the following items of the CES: *part of identity* [*F*(1) = 10.3, *p* = 0.002; η*_*p*_*^2^ = 0.079]; *comprehension* [*F*(1) = 7.28, *p* = 0.008; η*_*p*_*^2^ = 0.057]; *central in life* [*F*(1) = 9.27, *p* = 0.003; η*_*p*_*^2^ = 0.072]; *life change* [*F*(1) = 24.39, *p* < 0.000; η*_*p*_*^2^ = 0.169]; and *turning point* [*F*(1) = 10.01, *p* = 0.002; η*_*p*_*^2^ = 0.077]. With the covariate, all the experimental group’s means increased, whereas the control group’s ones decreased. Moreover, results showed a significant effect of groups on the item *life change*, after controlling both the *Need for Order* factor of the Need for Cognitive Closure Scale [*F*(1) = 22.62, *p* < 0.001; η*_*p*_*^2^ = 0.159] and the *Intolerance for Ambiguity* factor [*F*(1) = 24.61, *p* < 0.001; η*_*p*_*^2^ = 0.170]. After the *Mental Closure* factor of the Need for Cognitive Closure Scale was controlled for, results showed a significant effect of groups on these CES items: *Part of Identity* [*F*(1) = 9.13, *p* = 0.003; η*_*p*_*^2^ = 0.071]; *Comprehension* [*F*(1) = 5.76, *p* = 0.018; η*_*p*_*^2^ = 0.046]; *Central in life* [*F*(1) = 7.95, *p* = 0.006; η*_*p*_*^2^ = 0.062]; *Life change* [*F*(1) = 22.32, *p* < 0.001; η*_*p*_*^2^ = 0.157]; and *Turning point* [*F*(1) = 8.14, *p* = 0.005; η*_*p*_*^2^ = 0.064].

## Discussion

We assessed the effect of an ecological DE consisting in the format of the “dialogue in the dark” hosted by the Institute of Blinds in Milan on participants’ creative thinking dimensions of fluency, flexibility, elaboration, and originality. Drawing from existing theoretical and empirical evidence (e.g., Huang and Galinsky, 2011; Gocłowska et al., 2014, 2017, 2018), we tested the diversifying potential of this experience, and we identified the absence of light as a core violation. With this regard, a DE should be enough “stressing” to activate people’s resource while not exploiting them by turning into a threat instead of a challenge (Gocłowska et al., 2018). Indeed, “dialogue in the dark” resulted as a highly unusual and unexpected experience as well as highly personally impacting after 2 weeks, as measured by CES and its single components.

We reported a detailed description of the impact of “dialogue in the dark” on creative thinking, personal relevance, and affect in the following. We presented a final discussion linking all these aspects in the final part.

When people undergoing “dialogue in the dark” experience were compared with a group of individuals performing an equivalent experience but in the presence of light, regarding their creative thinking abilities, the control group outperformed those people undergoing “dialogue in the dark.” Despite that raters’ agreement was high and we controlled for potentially intervenient factors as openness to experience trait and need for closure, this difference in creativity in favor of the control group (vs. “dialogue in the dark”) still remained. A higher personal disposition to openness to experience increased the cognitive impact of the DE, in terms of experience’s comprehension, centrality in one’s life, feature as changing event, and turning point. On the other hand, a greater personal need for structure reduced the cognitive impact of the experience on both the experimental and control groups. Although this last result was in line with that of existing literature (e.g., Gocłowska et al., 2014, 2017, 2018), the detrimental effect of “dialogue in the dark” on creative thinking compared with that of the control group was not.

Conversely, the personal impact of “dialogue in the dark” group was significantly higher than that of the control group. The experimental group assessed the “dialogue in the dark” experience as significantly more engaging, unusual, unexpected, and extraordinary than the assessment given to the control experience by the control group. At the same time, the control group evaluated the experience in the park as significantly more ordinary. Participants in the experimental group performed poorly than did those in the control group. This result strengthened the diversifying nature of the selected “dialogue in the dark” group versus control group (Ritter et al., 2012; Ritter et al., 2014; Gocłowska et al., 2018). Moreover, despite that “dialogue in the dark” group and control group did not significantly differ regarding the global score of CES, the personally impacting nature of “dialogue in the dark” emerged at four main levels. “Dialogue in the dark” resulted as significantly more impacting at the level of identity, because participants felt that this experience had become a part of their personal identity more than did the control group. In addition, the experimental group felt that this experience changed their way of comprehending the world and others more than did the control group. Finally, the centrality of this experience resulted at the level of importance attributed to “dialogue in the dark” and the fact that it was perceived as a turning point more than did the control group.

At the level of affect, the “dialogue in the dark” experience group generated significantly more intense positive emotions than the control experience, thus supporting another asset of DEs, which is the positive perception associated with them, instead of that of threat (Gocłowska et al., 2018). No differences on NA and specific adjectives were found. This reassured regarding the affective similarity between the two experiences. “Dialogue in the dark” and control group experience differed only regarding PA, which was the main aim of an effective DE conductive of creativity. Participants of the experimental group self-reported higher levels of attention, enthusiasm, and concentration. Conversely, the control group felt more excited, nervous, irritable, and jittery.

To synthetize, results showed that despite that “dialogue in the dark” featured all the main assets of a creativity-conductive DE (i.e., unusualness, personal impact, positive perception instead of the negative one, and challenging nature), it did not enhance creativity assessed immediately after its occurrence.

It may be that “dialogue in the dark” was so intense that it induced a high cognitive and perceptual load, which, in turn, impacted their mental flexibility and creativity on the short term. Indeed, as Eimer (2004) claimed, for sighted people, vision dominates spatial perception, and the localization of events in external space involves a visually defined spatial reference frame (Eimer, 2004). Therefore, sighted people are impaired in the presence of conflicting external spatial information, and this may be linked to Dickerson and Kemeny’s (2004) framework: the response of an individual to a stressful condition depends on both the number of socio-evaluative threats and the number of uncontrollable elements present in the surrounding environment (Byron et al., 2010). Concerning the uncontrollability aspect, the dialogue in the dark experience deprived participants of their sight and, hence, of their accustomed reference frame. Therefore, it may have generated a condition of uncontrollability that, in turn, generated high stress in participants. This would be consistent with Mendes et al. (2007) findings, according to which individuals’ interaction with the unexpected (in their experiment: people who violated traditional stereotypes) leads to answers that are formulated through two evaluation components, identified by expectancy violation theory: (i) uncertainty and (ii) required effort (Bartholow et al., 2001). Therefore, it may be that the lack of familiarity and the uncontrollability of the event could have increased the uncertainty and required a high cognitive effort to make sense of the unexpected and unusual information (Bartholow et al., 2001). This would be consistent also with Bartholow et al. (2001), who asked participants to interact with partners who violated their expectations. These individuals were also more prone to employ greater cognitive and attention resources during these interactions. Also, Byron et al. (2010) claimed that too low or too high activation “can […] cause cognitive interference, which can hinder performance on cognitively demanding tasks” (Byron et al., 2010, p. 202).

These results may suggest two main aspects:

(i) The intense short-term impact of DE on people’s mental schema, thus hinting at a long-term benefit – in terms of creativity – associated with this kind of experiences. People would need more time to “elaborate” the experience and embody it into current mental schema or just to accommodate existing schema to expand the possibility to connect even far ideas.
(ii) A highly intense experience could negatively impact on people’s mental flexibility by overloading their cognitive resources to make sense of the violation itself.

## Conclusion

This is the first study proposing and succeeding in demonstrating the feasibility to investigate even complex DEs in a controlled way, thus outlining their creative and emotional profile. This study explored the effect of an ecological but controlled DE on people’s creative thinking. This research advanced previous studies by introducing a variant of DE with higher ecological validity. Moreover, in this study, the focus was on the schema-violation mechanism–creativity link, and also on the whole DE–creativity link. Although preliminary, this study showed that DEs are very complex phenomena and that they cannot be studied by being limited neither to their schema-violation mechanism – even if it is considered their core – nor to the experience built within the laboratory.

### Limitations and Future Directions

Preserving ecological validity and control at the same time is an enormous challenge. We maintained all the conditions as constant as possible expect for the dimension of presence versus absence of light, however, other factors could influence the final outcome. For instance, other personality measures, such as the Big Five Inventory, could act as moderators of the ecological DE–creativity link, as well as the cognitive effort perceived by participants immediately after the “dialogue in the dark” compared with the control one. In this regard, administering a cognitive task immediately after an intense and potentially stressful experience, as a DE should be, could have overloaded participants’ cognitive resources instead of nurturing them. Conversely, TTCT could have resulted as boring owing to the nature of the subtest we selected (i.e., unusual uses of a box). Therefore, as a future step, cognitive effort perceived by participants should be measured after they have completed the creativity task. In addition, a repeated assessment of participants’ creativity, after 1 week and 1 month, would be useful to understand whether allowing participants to recover from the experience and starting building upon it is beneficial for capitalizing on an ecological DE. Finally, another future step would regard the instruments that could be used. Other instruments could be used to measure DE’s impact, besides the CES, even though – owing to the potentially stressful nature of DEs and the exploratory aim of the study – this scale resulted as the most appropriate measure. Moreover, other instruments besides subtest 5 of the Torrance Tests could be used, maybe some measures less sensitive to the specific anticipatory task performed by participants (Glãveanu et al., 2019). Finally, although we replicated the recruitment method for both groups, our experience was not a well-established format as the “dialogue in the dark” experience; thus, this could be a potential limitation, despite that all groups were matched regarding all target variables.

## Data Availability Statement

The datasets generated for this study are available on request to the corresponding author.

## Ethics Statement

The studies involving human participants were reviewed and approved by Ethical Committee of the Università Cattolica del Sacro Cuore. The patients/participants provided their written informed consent to participate in this study.

## Author Contributions

AG, AC, and SC contributed to the conception and design of the study. SC collected the data in Study 1 and Study 2 and wrote the first draft of the manuscript. AC and SC organized the database, performed the statistical analysis, and wrote the final draft. SB and EG reviewed the final version of the manuscript. AG supervised the entire work. All authors contributed to the article and approved the submitted version.

## Conflict of Interest

The authors declare that the research was conducted in the absence of any commercial or financial relationships that could be construed as a potential conflict of interest.
